# Cell metabolism sets the differences between subpopulations of satellite cells (SCs)

**DOI:** 10.1186/1471-2121-14-24

**Published:** 2013-05-03

**Authors:** Andrea Repele, Ramona Lupi, Simon Eaton, Luca Urbani, Paolo De Coppi, Michelangelo Campanella

**Affiliations:** 1 Stem Cells and Regenerative Medicine Lab, Department of Woman and Child Health, University of Padua, Padua, Italy; 2 Institute of Child Health & Great Ormond Street Hospital, London, UK; 3 Department of Comparative Biomedical Sciences, the Royal Veterinary College, University College London; University of London, Royal College Street, London NW1 0TU, UK; 4 Consortium for Mitochondrial Research (CfMR), University College London; University of London, Royal College Street, London NW1 0TU, UK; 5 European Brain Research Institute, Rita Levi-Montalcini Foundation, 00140, Rome, Italy

**Keywords:** Satellite cells, Clones, Metabolism, CO_2_ production, Apoptosis

## Abstract

**Background:**

We have recently characterized two distinct populations of Satellite Cells (SCs) that differ in proliferation, regenerative potential, and mitochondrial coupling efficiency and classified these in Low Proliferative Clones (LPC) and High Proliferative Clones (HPC). Herewith, we have investigated their cell metabolism and individuated features that remark an intrinsic difference in basal physiology but that are retrievable also at the initial phases of their cloning.

**Results:**

Indeed, LPC and HPC can be distinguished for mitochondrial membrane potential (ΔΨ_m_) just after isolation from the fiber. This is matched by mitochondrial redox state measured via NAD^+^/NADH analysis and alternative respiratory CO_2_ production in cloned cells. All these parameters are accountable for metabolic differences reflected indeed by alternative expression of the glycolytic enzyme 6-phosphofructo-2-kinase/fructose-2,6-biphosphatase 3 (Pfkfb3). Also Ca^2+^ handling by mitochondria is different together with the sensitivity to apoptosis triggered via this pathway. Finally, according to the above, we were able to determine which one among the clones represents the suitable stem cell.

**Conclusions:**

These experimental observations report novel physiological features in the cell biology of SCs and refer to an intrinsic heterogeneity within which their stemness may reside.

## Background

Satellite cells (SCs) are indispensable for skeletal muscle fibers by determining their homeostasis via essential processes of repair [1]; thus defects in SCs lead to muscle pathologies [2-4].

SCs reside beneath the basal lamina of muscle fibers, representing 2-7% of the nuclei associated to a fiber [5,6]. Although mitotically quiescent, they become active by entering the cell cycle in response to several stimuli such as: stretch, injury or electrical stimulation. The descendants of activated SCs, called Myogenic Precursor Cells (MPCs), or myoblasts, undergo multiple rounds of division before fusion into newly formed or existing myotubes. Chargé and colleagues demonstrated that SCs are distinct from their daughter myogenic precursor cells by biological, biochemical and genetic criteria [7]. In this regard, crucial was the notion that activated SCs restore the resident SC pool [6-8].

SCs are unipotent stem cells with the unique ability to regenerate the skeletal muscle producing precursor cells. However, even though the pool of SCs is accepted as the major source of myonuclei in postnatal muscle, it is likely that SCs are not all multipotent stem cells, but present an intrinsic heterogeneity through which specific features of regenerative capacity may stand [9-11].

Thus, in the last years, it was clearly demonstrated that a dichotomy between differentiation towards osteogenic [12] or adipogenic pathway [4] does occur.

Evidences for diversity within the myogenic compartments have been indeed described both *in vitro* and *in vivo*[1]. Nonetheless, alternative sensitivity to high-dose irradiation revealed that at least two populations of SCs are present in rat’s skeletal muscle and recognizable by proliferative and myogenic capacities within a proportion that varies accordingly with the age [1]. A discovery as such may be of paramount importance as it could be instrumental to define endogenous read-outs useful to indicate: i) tissues tonicity, ii) intrinsic ability to withstand pathological conditions and iii) adaptive capacity during ageing.

We directly exploited this and did so in rat skeletal muscles within which we reported two subpopulations of SCs co-existing in fixed proportions on the single fiber and distinguishable predominantly for their proliferative capacity [13]. These two were consequently divided and named Low Proliferative Clone (LPC), with myogenic fate, and High Proliferative Clone (HPC), that spontaneously produced adipocytes.

In the same study we provided evidence that these two clones, although in the same pool, possess different myogenic gene expression and, quite interestingly, distinct metabolism for the differences in functional parameters for mitochondrial biology such as: i) mitochondrial membrane potential (ΔΨ_m_), ii) ATP production and iii) Reactive Oxygen Species (ROS) generation that renders the HPC much more glycolytic than the LPC.

### Hypothesis

The founding hypothesis of this work was that if subpopulations of SCs are characterized by distinctive metabolic profile, by outlining this we will define endogenous read-outs to distinguish muscle regenerating SCs populations.

### Aim

Aim of this study was to test this and to show such differences via imaging based techniques of cell physiology analysis in order to implement the tools on our disposal to construct strategies for efficient and unbiased recognition of precursor cells that guarantee the muscle homeostasis.

### Results

We have achieved this by profiling ΔΨ_m_, NAD^+^/NADH (Nicotinamide Adenine Dinucleotide) redox state, CO_2_ production, and glycolytic enzyme expression together with critical analysis of the mitochondrial buffering capacity for Ca^2+^[14] and cellular susceptibility to mitochondrial dependent apoptosis [15,16]. This was achieved in both cloned and uncloned cells to corroborate our analysis.

The outcome was a phenotypical cell metabolism that primes, precedes, and sustains the alternative capacity to enter the myogenic differentiation of SCs’ subpopulations.

## Results and discussion

**Figure 1:**
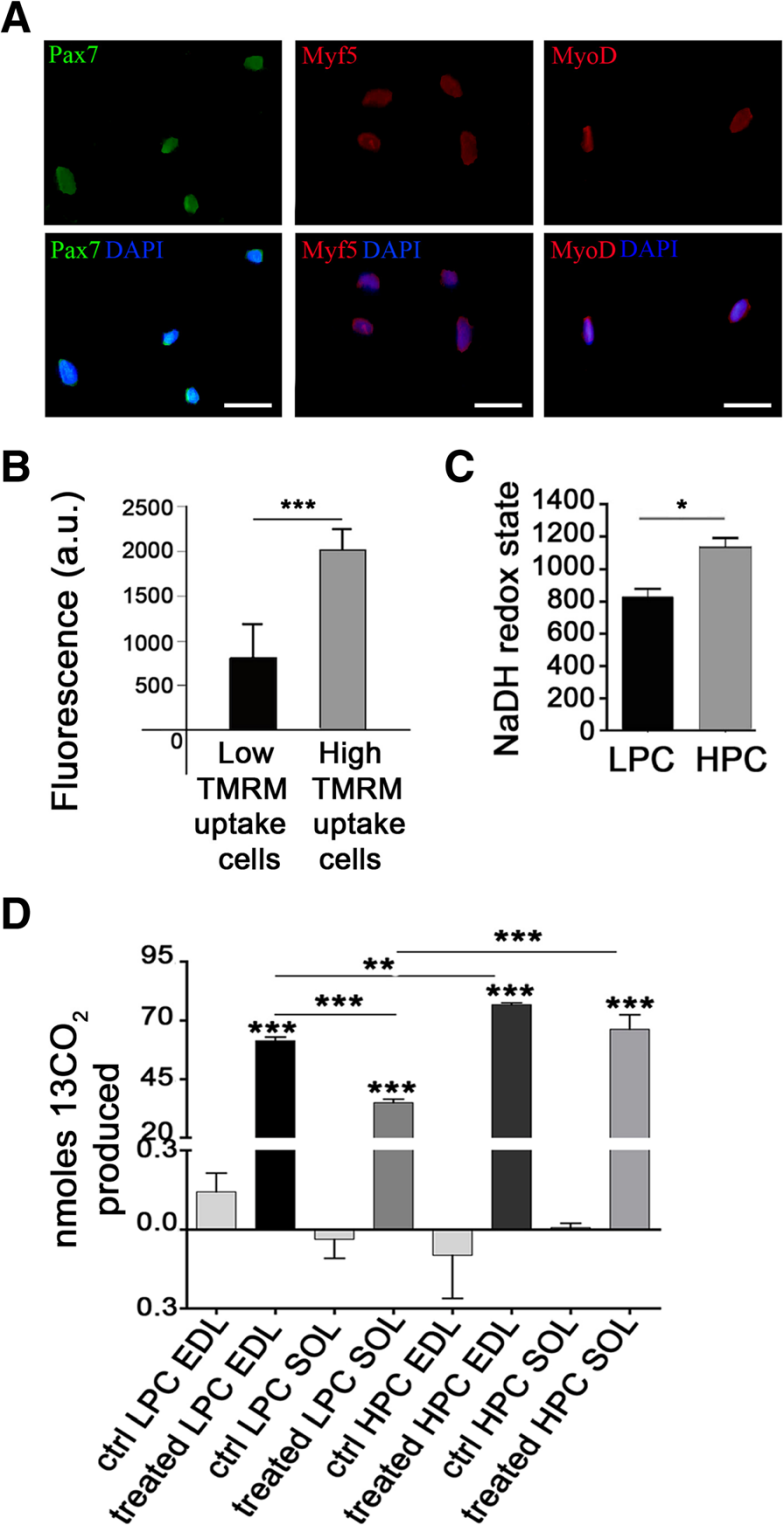
**Characterization of mitochondrial metabolism differences in both cloned and uncloned satellite cells. (A)** Freshly isolated SCs dissociated from single myofibers and seeded on gelatin-coated slides showed at immunofluorescence expression of satellite cell markers Pax7, Myf5 and MyoD (scale bar: 100 μm). **(B)** Time zero analysis: in 5 out of 5 preliminary experiments, after 24 hours of culture in muscle proliferating medium, SCs presented a different mitochondrial membrane potential ΔΨ_m_ (***p < 0.001). **(C)** Measurement of NaDH level: HPC and LPC demonstrated significant different redox states. These data confirmed that HPC has a glycolytic metabolism compared to low proliferative clones (*p < 0.05). **(D)** Measurement of CO_2_ after incubation of D-glucose U-C_13_ for 4 hours, in SC clones derived from fast and slow-twitch muscle fibers. Treated cells produced a higher amount of CO_2_ compared to untreated control cells (**p<0.01, ***p < 0.001). Furthermore, HPC showed higher CO_2_ production in respect to LPC, independently from the muscle type origin (***p < 0.001).

Following isolation of SCs from single fibers, we probed the cells for specific markers: Pax7, Myf5 and MyoD to confirm the phenotype (Figure 1A). Then we tested their homeostatic mitochondrial membrane potential (ΔΨ_m_) using the potentiometric dye tetramethyl rhodamine methyl ester (TMRM). TMRM was added on cells right after their isolation from the fiber and measuring the degree of dye uptake(Figure 1B). Interestingly, groups of cells within the same selection showed two distinct capacities to uptake TMRM that could be easily divided in one with a low (812 ± 398 arbitrary units, a.u.) and another one with high amount of loading for TMRM (2043 ± 362 a.u., ***p < 0.001) (Figure 1B). More interestingly, this was, in percentage terms, equal to that seen in the two clonal subpopulations [13], thus indicating that differences in mitochondrial performance are present at the very initial phases of the SCs’ life and in this way accounting for an inner heterogeneity before cloning and differentiation.

Subsequently, pools of SCs were cloned and divided into LPC and HPC on the basis of their growth ability [13]. LPC and HPC were therefore tested for their reduction oxidation potential, by monitoring the mitochondrial NAD^+^/NADH balance. This metabolic analysis does not require any exogenous dye since based on the auto-fluorescence of the mitochondrial pyridines via a dedicated UV laser [17]. The outcome of this is reported in Figure 1C and evidently shows the alternative state of mitochondrial oxidation. The normalized values reflect the state of resting respiration and originate from a standard experimental manoeuvre [18], in which the cells undergo pharmacological treatment with mitotoxins to sequentially induce maximal reduction (by inhibiting the respiration with NaCN) and oxidation (triggered by the un-coupler FCCP) to set the extremes of the respiratory performance and in this way extrapolate the starting levels [18]. This, as indicated in the plotted data, proved LPC and HPC to be opposite for “Redox State” with LPC more oxidised, hence mitochondria more coupled. The NAD^+^/NADH evaluation confirmed data on ΔΨ_m_ and emphasized that HPC do have a greater glycolytic metabolism compared to LPC (Figure 1C) (824.9 ± 104.9 and 1121.3 ± 141.7 a.u. respectively, *p < 0.05).

To further corroborate this evidence we checked another relevant parameter for cellular respiration assessing the level of carbon dioxide (CO_2_) generated by the two clones of SCs. To do so, LPC and HPC were kept in DMEM enriched of [U-^13^C]-D-glucose (25 mM) in absence of pyruvate and glucose. ^13^C-pyruvate produced by glycolysis is then oxidised in the mitochondria to yield ^13^CO_2_ so that ^13^CO_2_ is a measure of mitochondrial oxidative metabolism. After 4 hours of incubation with [U-^13^C]-D-glucose, we analysed by mass-spectrometer the ^13^CO_2_ (assessed in nmol) released in the culture medium and extrapolated the final values. These were then plotted and depicted as histogram in Figure 1D. It is clearly visible that CO_2_ produced by HPC is higher than LPC in both fast and slow twitch muscles tested for this experiment (HPC: 76.7 ± 1.8 EDL and 66.3 ± 15.1 *soleus*, LPC 61.3 ± 3.9 EDL and 35.0 ± 3.5 *soleus*, n=4, **p < 0.01, ***p<0.001), thus sustaining the theory that the HPC are more inclined to glycolytic respiration consequent to the depression of mitochondrial oxidation (Figure 1D).

To better exploit the metabolic phenotype, the expression of the glycolytic enzyme 6-phosphofructo-2-kinase/fructose-2,6-biphosphatase 3 (Pfkfb3) was profiled. During the cell cycle the energy and anabolic substrates must be efficient and tightly coupled to the regulation of metabolism and growth. The Pfkfb3 is a consolidated read out of this and was of our most direct interest since associated to pathological conditions such as human cancers and low oxygenated environment [19,20].

**Figure 2:**
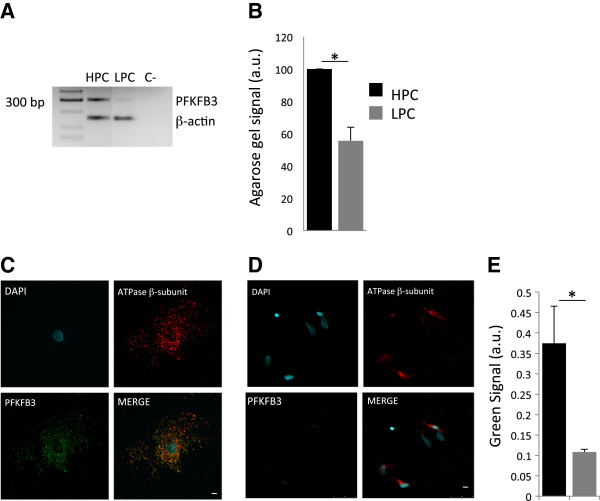
**Glycolytic enzyme analysis. (A)** RT-PCR for Pfkfb3 in HPC and LPC (housekeeping gene: β-actin). **(B)** Agarose gel signal between HPC and LPC (**p < 0.05). **(C)** Immunofluorescence for ATPase β-subunit and Pfkfb3 in LPC and **(D)** HPC (scale bar: 10 μm). **(E)** Intensity of Pfkfb3 green signal (**p < 0.05).

To run this assay, we retro-transcribed the mRNA of Pfkfb3 from both HPC and LPC and via RT-PCR recognized a higher level of this in HPC than in LPC (Figure 2A). This was confirmed statistically by normalizing to blank (LPC: 55 ± 8.33% of HPC, *p< 0.05) (Figure 2B) and the translation into protein was also investigated via immunofluorescence. This analysis –which was done by co-staining the cells with the β–subunit of the F_1_-Fo-ATPsynthase to mark size of the mitochondrial network- confirmed that the same proportion of enzyme is expressed with a greater quantity in HPC than in LPC (Figure 2C, D). The relative quantification (Figure 2E) confirmed that although both clonal types are efficient for this enzymatic pathway, HPC do have it in larger quantity (HPC: 0.37 ± 0.09 and LPC: 0.108 ± 0.006, *p < 0.05).

**Figure 3:**
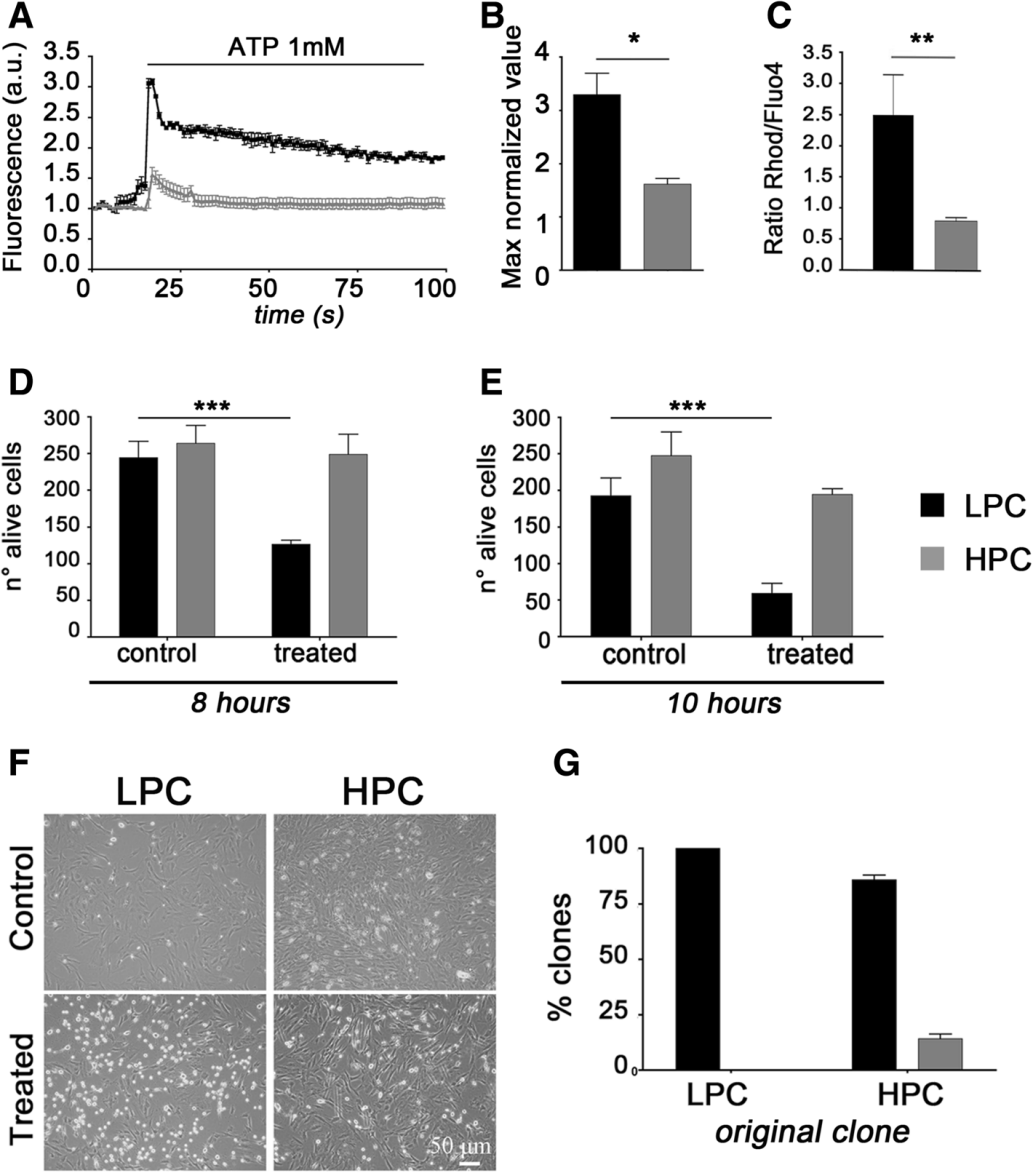
**Mitochondrial Ca**^**2+**^**analysis, ceramide sensitivity assay and subcloning test. (A, B)** Mitochondrial calcium level was followed in real time by measuring Rhod-5N dye. LPC and HPC were treated with ATP 1 mM and traces trend monitored over time. The diagram explains the maximum uptake of calcium in both clones (*p < 0.05). **(C)** Mitochondrial mass analysis showed statistical difference between HPC and LPC in term of the size of mitochondrial network (**p < 0.01). **(D, E)** C_2_-Ceramide (N-Acetylsphingosine) treatment after 8 and 10 hours respectively: the charts highlighted the number of cells before and after incubation with C_2_-Ceramide 20 μM in the clones. In both frames of time clones from LPC presented increased sensitivity to apoptosis than HPC (***p< 0.001). **(F)** Pictures of C_2_-Ceramide treated (8h) and untreated (control) cells (scale bar: 50 μm). **(G)** Sub-cloning of HPC and LPC demonstrating that only HPC could regenerate both clone types again.

The next step was to examine the cell signaling of HPC and LPC, by testing the level of mitochondrial Ca^2+^ accumulation important and additional feature to assess the physiological differences between these two sub-populations. HPC and LPC were loaded with the fluorescent dye rhodamine-5N, to measure [Ca^2+^]_m_[21,22] and, through live cell imaging, the uptake of Ca^2+^ by mitochondria was tracked after cells challenge with the IP_3_ generating stimulus ATP (1 mM) [15,23] (Figure 3A). The maximum level of Ca^2+^ uptake was recorded and calculated based on the peaks’ values that yielded the following outcome: higher mitochondrial Ca^2+^ uptake in LPC than in HPC (3.3 ± 0.7 and 1.6 ± 0.5 normalized value respectively) (Figure 3B).

Clones’ mitochondrial volume fraction was also extrapolated benefiting from a co-staining with the green fluorescent dye (Fluo4) for the [Ca^2+^]_c_ and the red fluorescent dye (Rhod-5N) for the [Ca^2+^]_m_. The ratio between the two signals indicated an increased mitochondrial mass in LPC (2.5 ± 0.7 and 0.80 ± 0.1 respectively) (Figure 3C), which is in line with a reduced mitochondrial activity.

In accordance with this, mitochondrial Ca^2+^ analysis suggested that a correlation was also likely with an alternative sensitivity to programmed cell death and this was tested using the Ca^2+^ dependent pro-apoptotic stimulus C_2_-Ceramide [24].

Clones were therefore treated with C_2_-Ceramide (20 μM for 8 and 10 hours) and the number of cells withstanding death counted and normalized for the total number of cells in each type of clone (Figure 3D and E). 8 hours of treatment produced in HPC substantial resistance to cell death as no difference occurs if compared with the untreated condition (248.8 ± 61.0 versus 264.0 ± 54.4 respectively p > 0.05). LPC showed instead a significant sensitivity to death compared with the untreated control (126.6 ± 12.3 versus 244.4 ± 49.5 respectively, ***p < 0.001) with identical outcome obtained after 10 hours of incubation (194.8 ± 16.2 versus treated LPC: 59.2 ± 30.8 n=4, ***p < 0.001). Representative images taken at time 0 and after 8h C_2_-ceramide treatment are reported in panel F of the same figure.

The resistance to intrinsic apoptosis is consequence of a reduced efficiency in the mitochondrial Ca^2+^ handling and denotes a phenotypic cellular physiology that likely associates with stronger proliferative ability as proven for every cell type with a marked glycolytic profile. The greater sensitivity to apoptosis of the LPC together with the greater buffering capacity for Ca^2+^ do sustain a metabolically active condition that fits with a myogenic fate that we already reported for the same type of cells [13].

If that was true the HPC would instead preserve a stem-like potential till the lineage commitment. To verify this hypothesis, each clone used for the above metabolic experiments was sub-cloned and analyzed after 10 days in culture. Notably, LPC gave rise exclusively to LPC sub-clones, whilst HPC showed the presence of both subpopulations in a ratio similar to what we already published [13] (85.9 ± 5.5% and 14.1 ± 5.5% versus 75% and 25% respectively) (Figure 3G).

This added evidence corroborates our initial hypothesis and emphasizes that the inner heterogeneity within SCs is based on a different predisposition to myogenic lineage. Nonetheless, this strongly argues that the stemness at the basis of tissues repairing may exclusively belongs to HPC in the face of LPC already committed.

## Conclusions

The activity of SCs permits skeletal muscle to relentlessly regenerate itself and in this way preserve tonicity and fitness. SCs are therefore granted of major attention in the community working in muscle biology and their physiological patterns, together with regenerative potential, are widely exploited. According to recent discoveries, muscle SCs constitute a heterogeneous population of muscle precursors, dominated by finely tuned niche-mechanisms, which play a peculiar role in its global balance. The complete understanding of these is therefore fundamental to solve some basic questions, but in particular to set up some clinical protocols to treat some of the related pathologies. The identification, as shown in this paper, of key underlining metabolic parameters at the basis of the SCs’ intrinsic diversity is a substantial contribution toward a more exhaustive comprehension of SCs’ physiology and a fundamental step to interprete their phsyiopathology and rationalize strategies of intervention.

## Methods

### Animals

Three to four month-old Sprague–Dawley wild type rats (Harlan, Indianapolis, U.S.A.) were used in this study. Animal care and experimental procedures were performed in accordance with “D.L. 27-1-1992, number 116, applicative declaration of Healthy Minister number 8 22-4-1994”. No *in vivo* experiments were run for this study.

### Isolation of single fibers from extensor digitorum longus and *Soleus* muscles

Single muscle fibers with associated SCs were isolated from *extensor digitorum longus* (EDL) and *soleus* (SOL) muscles. In brief, muscles were digested for 2 hours at 37°C in 0.2% (w/v) type I-collagenase (Sigma-Aldrich, St. Louis, MO), reconstituted in DMEM (high-glucose, with L-glutamine, supplemented with 1% penicillin-streptomycin, all from GIBCO-Invitrogen, Paisley, UK). Following digestion, muscles were transferred in plating medium (DMEM low-glucose, 10% HS, 1% penicillin-streptomycin, 0.5% chicken embryo extract, all from GIBCO-Invitrogen) and gently triturated with a wide bore pipette to release single myofibers. In each preparation, under phase contrast microscope, single fibers were carefully transferred in a 10 cm-plate containing 10 ml of muscle plating medium (1° dilution). Each single fiber was subsequently transferred in another 10 cm plate containing 10 ml of muscle plating medium (2° dilution). Finally, each fiber was collected into one 50 ml Falcon tube with 1 ml of muscle proliferating medium (3° dilution in DMEM low-glucose, 20% FBS, 10% HS, 1% penicillin-streptomycin, 0.5% chicken embryo extract). Serial dilution was performed in order to avoid the presence of contaminant cells.

### Cloning satellite cells from single myofibers

Clones of satellite cells were derived from EDL and SOL myofibers. After dilution, single fibers were triturated 20 times using a 18 G needle mounted onto a 1 ml syringe, to disengage SCs. The resulting cell suspension was diluted with proliferating medium and then dispensed into 96-well petri dishes with limiting dilution (0.5 cell/well). Dishes were incubated at 37.5°C, 5% CO_2_ in a humidified tissue culture incubator. Clones were counted after 5 and 10 days with inverted-microscope analysis and Bürker counting chamber.

### Imaging ΔΨ_m_

Tetramethyl rhodamine methyl ester (TMRM, 50 nM, Invitrogen, Paisley, UK) was used in “redistribution mode”: the dye was allowed to equilibrate and was present continuously. The TMRM fluorescence intensity was quantified by removing background signals by “thresholding” and measuring the mean fluorescence of the pixels contained in mitochondria. Thus, the signal is independent of mitochondrial mass and only reflects the dye concentration within individual mitochondrial structures.

### RT-PCR

Total RNA was isolated from HPC and LPC using Trizol (Invitrogen). Quantity and integrity of each samples was checked using Agilent BioAnalyzer 2100 (Agilent RNA 6000 nano kit). Three different aliquots of HPC and LPC RNA sample were retro-transcribed using GoScript™ Reverse Transcription System (Promega, Madison, WI, USA) following manufacturer instructions. Oligonucleotides used to amplify Pfkfb3 cDNA were previously described [19]. Amplification was conducted using the following conditions: 4 minutes at 95°C; 95°C for 30 sec, 60°C for 30 sec, 72°C for 30 sec (35 cycles); final extension was carried out for 7 min at 72°C.

### Immunofluorescence analysis

Immunofluorescence staining was conducted on HPC and LPC fixed in PFA 4%. Cells were permeabilized with 0.01% Triton X-100 (Sigma-AldrichSt. Louis, Missouri, USA) in PBS (GIBCO-Invitrogen, Carlsbad, CA, USA) for 1 min and blocked in BSA 1% (Sigma-Aldrich), PBS pH7.5 for 60 minutes. Primary antibodies used were: mouse anti-ATPase b subunit (diluted in blocking solution 1:1000; Abcam, Cambridge, UK) and goat anti-Pfkfb3 (1:100; Santa Cruz Biotechnology, Santa Cruz, CA. USA). Secondary anti-mouse and anti-goat antibodies (1:500; Alexa Fluor, Molecular Probes, Eugene, Oregon, USA) were used. Nuclei were stained with 0.01% 4,6-diamino-2-phenylindole HCI (DAPI, Applichem, Darmstadt, Germany) and the coverslips were mounted using Prolong Gold antifade reagent (Molecular Probes, Invitrogen). Negative controls were performed by omission of primary or secondary antibodies.

### NADH measurement

Mitochondrial “redox state” was measured via imaging assessment of the NADH auto-fluorescence as per protocol reported in [18].

### Ca^2+^ and cell death analysis

Coverslips were incubated with Rhod-5N dyes (10 μM, Molecular Probes, Eugene, Oregon, USA) to label the mitochondrial network. All fluorescent images were captured on Zeiss 510 LSM confocal microscope equipped with a 40X oil-immersion lens. For apoptosis treatment, cells were incubated for 8 and 10 hours with C_2_-Ceramide (20 μM) to determine the number of living cells.

### CO_2_ measurement

CO_2_ production was used as a marker of mitochondrial oxidative metabolism. Two different types of culture media were employed: DMEM (GIBCO-Invitrogen) which contains 4.5 mg/l (25 mM) D-glucose (Invitrogen), sodium pyruvate (Invitrogen), sodium bicarbonate (NaHCO_3_, Sigma Aldrich) (44 mM) and DMEM with NaHCO_3_ (44 mM) and no glucose, supplemented with 25 mM of [U-^13^C] D-glucose (Cambridge Isotope Laboratories, Inc., Andover, MA, USA). Cells were cultured in hermetic screw caps 25 cm^2^ flasks (Corning – Sigma Aldrich) at 1000 cells/cm^2^. Media supplemented with [U-^13^C] D-glucose was substituted to culture media and flasks were sealed for four hours. Following this, 500 μl culture medium was transferred to a 12 ml glass tube (Exetainer, Labco, UK) and glacial acetic acid (100 μl) was injected through the septum to release CO_2_ from bicarbonate. ^13^CO_2_/^12^CO_2_ was measured in the gas phase by isotope ratio mass spectrometry. The ratio of ^13^CO_2_ to ^12^CO_2_ produced is proportional to the quantity of oxidized glucose [25].

### Statistical analysis

Data are presented as mean ± s.d. Comparison between groups used the t-test and ANOVA assuming two-tailed distribution, with an alpha level of 0.05.

## Competing interests

The authors declare no competing interests.

## Authors’ contributions

Conceived and designed the experiments: AR, PDC, MC. Performed the experiments: AR, RL. Analyzed the data: AR, RL, PDC, MC. Contributed reagents/materials/analysis tools: SE, PDC, MC. Wrote the paper: AR, MC. All authors read and approved the final manuscript.
